# Effects of Bee Pollen on Growth Performance, Intestinal Microbiota and Histomorphometry in African Catfish

**DOI:** 10.3390/ani13010132

**Published:** 2022-12-29

**Authors:** Joanna Nowosad, Szymon Jasiński, Martyna Arciuch-Rutkowska, Hany M. R. Abdel-Latif, Marcin Wróbel, Mateusz Mikiewicz, Łukasz Zielonka, Ihor Y. Kotsyumbas, Viktor P. Muzyka, Oksana M. Brezvyn, Grzegorz Dietrich, Dariusz Kucharczyk

**Affiliations:** 1 Department of Research and Development, Chemprof, 11-041 Olsztyn, Poland; 2 The Stanislaw Sakowicz Inland Fisheries Institute, 10-719 Olsztyn, Poland; 3 Department of Ichthyology and Aquaculture, Warmia and Mazury University in Olsztyn, 10-701 Olsztyn, Poland; 4 Department of Poultry and Fish Diseases, Faculty of Veterinary Medicine, Alexandria University, Alexandria 22758, Egypt; 5 Department of Veterinary Prevention and Feed Hygiene, Faculty of Veterinary Medicine, University of Warmia and Mazury in Olsztyn, 10-719 Olsztyn, Poland; 6 Department of Pathological Anatomy, Faculty of Veterinary Medicine, University of Warmia and Mazury in Olsztyn, 10-719 Olsztyn, Poland; 7 State Scientific-Research Control Institute of Veterinary Preparations and Feed Additives, 79000 Lviv, Ukraine

**Keywords:** *Clarias gariepinus*, honeybee pollen, intestinal morphology, lactic acid bacteria, nutrition, prebiotics

## Abstract

**Simple Summary:**

African catfish is one of the most perspective fish species in modern aquaculture. However, the main cost involved in its production is focused chiefly on the cost of feeds. The main reason for this is the behavior of this species (predatory fish) and the high rate of cannibalism in intensive culture. That is why it is essential to assess functional bioactive additives to be used in commercial feed with a positive effect on the growth and survival rates of this commercially valued fish species. In the present study, African catfish juveniles were fed diets enriched with honeybee pollen (BP) and were tested for growth, survival, intestinal microbiota, and tissue histomorphology. It has been shown that BP addition positively affected all the analyzed parameters. This research study spotlights the benefits of BP as a prebiotic supplement for improving the functionality of feeds prepared for African catfish.

**Abstract:**

This study aimed to determine the dietary effects of honeybee pollen (BP) on growth parameters, intestinal microbiota, hepatic histoarchitecture, and intestinal histomorphometry of African catfish *Clarias gariepinus* juveniles. The feeding experiment was carried out in a recirculating aquaculture system under controlled conditions for 21 days to achieve more than a 10-fold increase in weight in fish from the control group. Fish were fed well-balanced commercial feed without any supplements and served as a reference group (group C) and other diets enriched with varying BP levels as 1% (BP1), 2% (BP2), and 3% (BP3). Results showed a significant (*p* < 0.05) effect of the dietary BP not only on the growth parameters (such as final body weight: 5.0 g to 6.6–7.5 g, weight gain: 0.23 g/d to 0.31–0.35 g/d, body length: 84.7 mm to 93.8–95.9 mm, and specific growth rate: 11.7%/d to 13.1–13.7%/d, group C vs. experimental groups, respectively) but also on the development of beneficially important gut microbiota, such as lactic acid-producing bacteria. In BP-enriched groups, an average of 45% higher body weight gain was observed compared to those reared in the control group. The histological analysis showed that dietary BP may have a positive effect on the development of the intestinal tract and may enhance the absorption of nutrients with the potential ability to maintain a normal hepatic histoarchitecture of the treated African catfish. The results obtained suggest the optimum level of BP additive to feed for African catfish should be 1%.

## 1. Introduction

The continuous demand for fish and seafood means global aquaculture production continues to grow. It is well-known that food from aquaculture is not only recognized as a source of highly digestible protein but also for essential Ω-3 fatty acids and micronutrients. The development of aquaculture forces fish farmers to increase breeding rates by improving new protocols for culturing commercial fish species or introducing new species to aquaculture [1]. In just two decades (1997–2017), the total aquaculture production tripled to 112 metric megatons in 2017 [2]. For this reason, aquatic foods are increasingly recognized for their crucial role in food biosecurity and nutrition [1].

One of the fastest growing production volumes of freshwater fishes is African catfish (*Clarias gariepinus*) [1,3]. The total production of this fish species is steadily increasing and spreading worldwide [4,5]. According to FAO [1], the production of African catfish in 2020 amounted to 1249 thousand metric tons, estimated to be about 2.5% of the total finfish production. The popularity of African catfish on the world market is increasing due to its tasty, highly valuable, nutritious meat [6] and its fast weight gain. This fish species reaches commercial size even 6–8 months after hatching [7]. In addition, it is relatively resistant to extreme environmental conditions (e.g., oxygen deficiency, high concentrations of ammonia, and nitrites) [8,9]. It can be reared in high densities of up to 400–700 kg per 1 m^3^ [10]. Although the African catfish has a good feed conversion factor [11], the highest costs related to its production are focused principally on the feed cost. The calculation for a theoretical RAS facility, which would produce 500 metric tons of African catfish annually at densities of 230 kg per 1 m^3^, indicates that feed costs account for 66% of all costs incurred [12]. According to the data obtained from Thailand aquaculture, the costs of feed used for culturing catfish hybrids *Clarias gariepinus* × *C. macrocephalus* in both intensive and semi-intensive systems account for over 80% of the total expenses [13]. This is one of the highest recorded cost levels in relation to total costs in intensive aquaculture.

Feed for juvenile (larvae, fry) African catfish should contain both high level of crude protein and crude fat. The nutritional value of the feed should be adapted to the changing needs of fish resulting from their size and rearing conditions [14]. On the other side, due to the legal restrictions on the use of antibiotics as growth promoters because of their negative impacts on the environment and the consumers, scientific work should be undertaken on a wider scale of feed additives to find suitable, environmentally friendly, and safe alternatives [15]. Including several non-antibiotic growth promoters in the diet is not a new issue; however, the multitude of substances with potentially beneficial effects and the ever-changing needs of the aquaculture sector require further research. This fish species has increased cannibalism [5], which may result from failure to meet the nutritional requirements, especially vitamins [16,17]. Moreover, it was reported that the survival rate of catfish fries up to the first two months of life is often around 60% or less [18,19]. Therefore, it is necessary to use antibiotic alternatives to increase fish survivability and enhance their resistance and immune responses. Currently, various biologically active substances contained in plants or herbs and other medicinal preparations of natural origin, including honey bee pollen and propolis, are being tested as feed additives for fish and aquatic invertebrates [20,21,22,23]. The positive effect of these additives on growth and growth parameters, survival, resistance to stress and diseases, development of the intestine and its microbiome, reproductive performance, fillet quality and many others has been described many times [24,25,26,27].

Honeybee pollen (BP) is rich in many essential nutrients such as fatty acids, amino acids, vitamins; therefore, it can be a useful supplement to fish diet [24,25,26]. It is one of the bee products produced by the honeybee (*Apis mellifera*). During collection, the honeybees enrich it with about 10% honey and then collect it on their legs, creating characteristic granules known as “bee pollen” [28]. The most significant compounds in BP are carbohydrates (13–78%), representing both polysaccharides and simple sugars (mainly glucose and fructose). It also contains 10–40% protein, which consists of simple proteins and free amino acids. Its crude fat content is found primarily in glycerolipids and fatty acids (mainly palmitic, linoleic, α-linolenic, and oleic acids) and ranges from 1–13%. It is also rich in numerous elements, including K (2353–20,000 mg/kg), Mg (200–3000 mg/kg), P (800–9590 mg/kg), and Na (9–846 mg /kg) and Ca (200–3050 mg/kg) which constitute about 10% of the total pool of the mineral compounds. Vitamins and provitamins present in BP in the highest concentrations are ascorbic acid (70–560 mg/kg), α-tocopherol (40–320 mg/kg), and β-carotene (10–200 mg/kg). BP is also rich in polyphenols, flavonoids, phytosterols, and other health-promoting substances [29,30,31,32,33]. 

Studies on BP use in animal nutrition have been carried out using both warm- and cold-blooded organisms. In mammals and fish production, it has several beneficial impacts on reproduction and health [34,35,36]. With a particular concern on aquaculture studies, earlier studies have shown that dietary supplementation BP enhanced the growth, immunity, and disease resistance in Nile tilapia (*Oreochromis niloticus*) [37] and in rainbow trout (*Oncorhynchus mykiss*) [38,39]. The BP extract also improved the immune responses of gilthead seabream (*Sparus aurata*) [24]. Moreover, dietary BP positively influenced immunity and disease resistance [25], as well as the composition of the intestinal microbiota of zebrafish (*Danio rerio*) [23]. Conversely, Panettieri et al. [40] found that dietary BP negatively affected the growth performance and nutrient digestibility of meagre (*Argyrosomus regius*) juveniles. To our knowledge, no studies have been published on the dietary uses of BP in African catfish diets. In addition, studies conducted on fish fry fed with biologically active additives such as propolis or honey bee pollen are relatively rare. Thus, this study aimed to determine the effect of adding BP to feed on the growth performance, survival rate, and body composition of African catfish during rearing under controlled conditions. In addition, histological analysis of the liver and intestine and a quantitative assessment of the composition of the intestinal microflora have also been examined.

## 2. Materials and Methods

### 2.1. Research Material and Studied Animals

The experiment was carried out at the Center for Aquaculture and Ecological Engineering in Olsztyn (Department of Aquaculture and Fisheries, Faculty of Animal Bioengineering, Warmia and Mazury University in Olsztyn, Poland). The material used for the study was obtained by artificial reproduction of the African catfish, which was conducted under controlled conditions according to the methodologies described by Abdel-Latif et al. [41] with the modification of fertilization described by Kucharczyk et al. [3] and Kucska et al. [42]. Eggs from two females (average weight: 3.2 ± 0.3 kg) and sperm from three males (average weight: 2.6 ± 0.8 kg) were used for fertilization. Eggs were incubated in Weiss jars at 24.5 ± 0.1 °C. The hatched larvae were reared at 25.0 ± 0.1 °C and were fed three times a day ad libitum with freshly hatched brine shrimp nauplii (*Artemia* sp., Ocean Nutrition, UT, USA). After two weeks, the larvae were given artificial food. Six hundred juveniles of African catfish (*n* = 600; 26 DPH, days post-hatching) with an average initial weight and body length of 0.48 ± 0.17 g and 38.9 ± 4.5 mm, respectively, were used for the study.

### 2.2. Feed Preparation 

In the experiment, a commercial feed with granulation of 0.9–1.6 mm was used (composition: crude protein 62%, crude fat 12%, carbohydrates 6.2%, cellulose 0.8%, crude ash 13%, phosphorus 1.4%, and sodium 0.9% (Aller Aqua, Christiansfeld, Denmark). Currently, commercial feeds dedicated to juvenile African catfish have a crude protein level of 58–62%. The basic feed for the research was chosen deliberately with high crude protein content so that low a protein level would not affect survival, mainly the cannibalism levels [16]. Fish from the control group (group C) were fed commercial feed supplemented with a mixture of rapeseed oil and water (1:1; 25 mL: 25 mL) in 50 mL per 1 kg of feed. Fish from experimental group (BP1, BP2, BP3) were fed the commercial supplemented with a mixture of rapeseed oil and water (same as the control group) enriched additionally with the appropriate amount of BP (1%, 2% or 3%). To prepare the feed for the control group, distilled water and rapeseed oil (1:1; together 50 mL kg^−1^ of the feed) homogenized and sprayed onto the feed. To prepare the feed for the research groups, the appropriate amount of BP was dissolved in distilled water (25 mL kg^−1^ of the feed), the same amount of rapeseed oil (25 mL kg^−1^ of the feed) was added. Then, the whole ingredients were homogenized and sprayed onto the feed. The feed was dried for 24 h at room temperature.

### 2.3. Experimental Design, Fish Management, and Sampling Procedures

Healthy fish were divided into 4 research groups of 150 fish each (50 fish/20 dm^3^ tank; in triplicate), which were fed with: commercial feed-control group (group C or 0% BP); commercial feed supplemented with 1% (group BP1), 2% (group BP2) and 3% (group BP3) of the bee pollen. Fish were reared for 21 days at 25 °C. The duration of the experiment was planned until the weight increased at least 10 times in the fish from the control group. Tanks were placed in a closed water recirculation system (RAS). The photoperiod was kept constant at 12 h light: 12 h dark. The water flow were 600 mL/min. Throughout the experiment, water parameters were monitored, such as: oxygen saturation (above 80%), pH (8.1 ± 0.1), nitrite and ammonia (both below 0.1 mg dm^−3^), using a handheld oxygen meter and handheld pH-meter (OxyGuard, Farum, Denmark) and using a DR 5000 spectrophotometer (HACH LANGE, Berlin, Germany) and cuvette tests: nitrite nitrogen (LCK 341) and ammonia nitrogen (LCK 304) [43].

Fish were hand-fed 4 times a day at 8:00, 11:00, 14:00 and 17:00 until apparent satiety. The tanks were cleaned, and dead individuals were removed and counted twice daily (before morning and evening feedings). Every 7 days and at the end of the experiment, fish body weight and length measurements were made. For this purpose, 10 random individuals were collected from each aquarium (*n* = 30 form each group) and were anaesthetized in a solution of buffered MS-222 (Finquel, Los Angeles, CA, USA) in a dose of 0.15 g dm^−3^, then weighed (±0.001 g) and measured (±0.1 mm) with an electronic digital caliper (Artykuły Techniczne "Tar-G", Radom, Poland). After the measurements, the fish were returned to the appropriate tanks. After the experiment, 5 fish from each tank were caught from each aquarium (*n* = 15 from each group) and then euthanized by bathing in MS-222 (dose 0.2 g dm^−3^). Samples for the study of the intestinal microbiota (*n* = 8 from each group), histological analysis (*n* = 7 from each group), and body composition analysis (*n* = 7 from each group) were collected for further analysis.

### 2.4. Rearing Indicators and Equations

The calculations used to evaluate the survival rates and growth are:

Cumulative natural mortality (CNM) (%) = [N_m_ × 100%]/N_i_, where: N_i_—number of fish at the beginning of the experiment; N_m_—number of fish natural died

Weight gain (g/d) = [BW_f_ – BW_i_]/T, where: BW_i_—initial fish weight [g], BW_f_—final fish weight [g], T—time of duration experiment (d)

Specific growth rate (SGR; %/d) = [lnBW_f_ – lnBW_i_] × 100% T^−1^, 

Fulton condition factor (K) = 100 × [BW_f_ × L_f_^−3^], where: L_f_ is body length (cm),

Relative growth rate (RGR; %) = [(BW_f_ – BW_i_)/BW_i_] × 100% [44].

At the end of the experiment, the fish present in each aquarium was counted. These data were used to calculate the cannibalism coefficient (CF) as follows:

CF (%) = N_f_/N_i_ × 100%, where: N_f_—the number of fish left in the tank at the end of the experiment.

Total survival rate (TSR%) = [N_i_ – (CNM + CF)]/N_i_ × 100%.

### 2.5. Origin of Bee Pollen

The bee pollen (BP) used in the present study was provided from a honeybee farm (Dębowa Łęka, Poland). It was a dried material and met the requirements of the non-obligatory standard PN-R-78893:1996 (Bee pollen). Based on the organoleptic evaluation of the honeybee pollen and the knowledge of the harvesting period, they were defined as honeybee pollen obtained mainly from rapeseed (*Brassica napus*) crops.

### 2.6. The Chemical Composition of BP used in the Present Study

#### 2.6.1. Extract BP Analysis 

The crude BP grains were milled for 2 minutes using a commercial grinder (First Two Tops). Each sample (1.0 g) of the finely ground BP was extracted using 70% ethanol in water. The extraction was carried out over 1 h in 14 mL of solvent using a vortex mixer (Corning model 6776, Merck KGaA, Darmstadt, Germany) at room temperature. The next sonication extraction stage was performed using a sonicator (Hielscher model UP100H, Hielscher Ultrasonics GmbH, Teltow, Germany). The amplitude was set at 100%, and the cycle was set at 1. The extraction process was carried out for 5 min in an ice bath. In the next step, the sample was centrifuged for 10 min at 6000 rpm. The supernatant was separated, the solvent was replaced with fresh solvent, and the extraction process was repeated twice. Filtrates were combined and made up to 50 mL with the respective solvent and then stored for one day at −20 °C until analyzed. Before analysis, the obtained extract was filtered (syringe filters, 0.45 µm, nylon) and twenty times diluted.

#### 2.6.2. Determination of the Total Antioxidant Capacity (TAC)

The total antioxidant capacity (TAC) of hydro-ethanolic BP extract was evaluated by using the DPPH free radical scavenging assay as described by Lawag et al. [45] with modifications. Briefly, 100 µL of the BP extract or Trolox standards were placed in a test tube, followed by the addition of 250 µL of the DPPH solution and 1500 µL 80% methanol in water. The reaction mixture was kept in the dark for 30 min at room temperature, and absorbance was read at 520 nm (UV-VIS Perkin Elmer Lambda 265 spectrophotometer, Perkin Elmer Shared Services, Kraków, Poland). The mean free radical scavenging activity of triplicate samples was expressed as the Trolox equivalent (TE) as mM Trolox or mg Trolox per g of pollen and IC_50_ (mg/mL). The DPPH reaction mixture was used at a concentration of 0.130 µM. Trolox in a concentration range of 12–60 µM was used as the standard.

#### 2.6.3. Determination of Total Flavonoid Content (TFC)

Quercetin was used as a standard, and the results were based on the standard curve equation of quercetin (1.1–34.0 µg/mL) and expressed as quercetin equivalent (mg QE) per g of the BP extract. The total flavonoid content (TFC) was determined based on the method by Zielińska and Turemko [46] with some modifications. A 1230 µL of diluted BP extract was mixed with 62 µL of 5% NaNO_2_ solution (*m/v*) in a test tube. After incubation at room temperature for 6 min, 123 µL of 10% AlCl_3_·6H_2_O was added. Again, the mixture was incubated under the same conditions for 6 min, then 410 µL of 1M NaOH was added, and the mixture was centrifuged for 10 min at 2000 rpm (Mini Spin Plus, Eppendorf; Germany). The absorbance of the reaction mixture was measured against the reagent blank at 510 nm with the spectrophotometer (UV-VIS Perkin Elmer Lambda 265, Perkin Elmer Shared Services, Kraków, Poland).

#### 2.6.4. Determination of Total Phenolic Content (TPC)

A dilute Folin-Ciocalteu reagent was prepared by mixing 1 mL of Folin–Ciocalteu reagent with 30 mL deionized water. A gallic acid standard solution ranging in concentration from 6.8 µg/mL to 34.1 µg/mL was prepared by dissolving in deionized water. The TPC assay was performed based on the methodology described by Lawag et al. [45] with minor modifications. In brief, for the analysis, 200 µL of pollen extract or 200 µL of gallic acid standard solution were placed in a test tube followed by the addition of 1 mL of the diluted Folin–Ciocalteu reagent. The mixture could react for 5 min. After this time, 800 µL of 0.75% Na_2_CO_3_ water solution were added. Samples were left to stand for 2 h at room temperature in the dark and then absorbance was read at 760 nm (UV-VIS Perkin Elmer Lambda 265 spectrophotometer) using 200 µL of 70% ethanol in water with other TPC reagents as a blank. The analysis was carried out in triplicate. The antioxidant activity was expressed as mg of gallic acid equivalent (mg GAE) per g of pollen, using the linear regression gained from the gallic acid calibration curve.

### 2.7. Chemical Analysis of Elements found in the Fish Body

Mineralization of the samples was carried out in Teflon vessels with 10 mL of nitric acid (69–70%; Baker Instra-Analyzed Reagent, Phillipsburg, NJ, USA). Mineralization was carried out using a Titan Microwave Digestion Systems MPS (Perkin Elmer, Shared Services, Kraków, Poland). Parameters during mineralization in four cycles (I-IV) during 70 min together (respectively: temperature, pressure, duration I: 170 °C, 30 bar, 5 min; II: 190 °C, 35 bar, 10 min; III: 200 °C, 35 bar, 20 min; IV: 50 °C, 30 bar, 25 min). Chemical analyses of elements present in the muscular tissue of treated African catfish were performed to analyze calcium (Ca), zinc (Zn), manganese (Mn), iron (Fe), potassium (K), sodium (Na), and magnesium (Mg). The elements were analyzed using Inductively Coupled Plasma Optical Emission Spectrometry procedures (ICP-OES, Perkin Elmer Shared Services, Kraków, Poland). The preparation of samples for ICP analysis was conducted using the microwave sample preparation system for pressure digestion. The analysis was performed using ICP-OES procedures (Avio 220 ICP-OES, Parkin Elmer Shared Services, Kraków, Poland).

### 2.8. Intestinal Microbiota

Before intestinal sampling, the fish body surface was decontaminated with a 70% isopropanol solution. The intestines were dissected under sterile conditions under a lamina flow chamber (Nordic Safe ESCO II BSC, Waltham, MA, USA) by using previously sterilized instruments. The intestinal content was placed in sterile 2 mL microcentrifuge tubes (Eppendorf type) and stored for further analysis at −20 °C. In the next step, the intestinal content was homogenized and plated in duplicate on MRS (de Man, Rogosa and Sharpe, Graso Biotech) medium to determine the total count of microorganisms and the number of lactic acid bacteria (LAB). The plates were incubated at 30 °C and 37 °C for 48 h, respectively (Memmert In110 Incubator, Memmert GmbH + Co. KG, Schwabach, Germany). After incubation, the grown colonies were counted, and the characteristic units on the MRS medium were subjected to MALDI-TOF analysis for accurate strain identification modified method from Kühlwein et al. [47].

### 2.9. Histological Analysis 

The sections obtained from liver and middle intestine samples were fixed in Bouin’s fluid. The fixed material was put into biopsy cartridges and then into a tissue processor (LEICA TD 1020, Wetzlar, Germany) for 21 h, where it was washed in ethanol at increasing concentrations (75, 80, 90, and 95%), acetone, xylene, and liquid paraffin at 54 °C. The obtained study material was sealed in paraffin blocks and sliced in a rotating microtome (LEICA RM 2155, Wetzlar, Germany) into 6–7 µm thick sequences. The paraffin sections were put onto protein-covered slides. The preparations were made with Mayer’s hemotoxin and eosin stain [48]. Subsequently, the stained preparations were sealed with coverslips and histokitt (Glaswarenfabrik Karl Hecht GmbH & Co KG, Sondheim vor der Rhön, Germany). The histological preparations were analyzed under a light microscope (Olympus BX51, Tokyo, Japan) with Cell^D software (Olympus UK Ltd., Hertfordshire, UK).

### 2.10. Statistical Analysis

Data are presented as means ± standard deviation (SD). Statistical differences among the groups were assessed by one-way ANOVA analyses, followed by Tukey’s test. The normality of the variables was confirmed by the Shapiro–Wilk’s test and the homogeneity of variance by Levene’s test. The results were considered statistically significant when *p* < 0.05. All the data were analyzed by Statistica (version 13.3, TIBCO Software Inc., Palo Alto, CA, USA).

## 3. Results

### 3.1. Effects of BP on the Fish Rearing Indicators

Statistically significant differences (*p* < 0.05) in the mean weight and length of the fish bodies compared to the control group were found in all groups fed BP-supplemented diets (Table 1). At the end of the experiment, the control group (group C) had an average weight of 5.0 ± 2.1 g and a total length of 84.7 ± 9.7 mm. Growth in this group was 0.23 g/d, and SGR was 11.7%/d (Table 1). These parameters were statistically significantly lower (*p* < 0.05) than those recorded in the study groups BP1, BP2, and BP3 (Table 1). Although there were no statistically significant differences between the test groups (BP1, BP2 and BP3 groups), the highest values of the analyzed parameters were obtained in the BP3 group. The values of the condition coefficient of the individual groups did not significantly differ among the experimental groups. Both stock survival and cannibalism showed no significant differences between groups. The highest natural mortality of over 11% was observed in group C. The percentage of cannibalism in this group was also the highest and amounted to 10%; however, these values were not statistically significant compared to the other groups (Table 1).

### 3.2. Analysis of TAC, TFC and TPC of BP

This study showed that BP, used as a feed supplement for juvenile African catfish, contained 19.64 mg/g total flavonoid content (TFC) and 28.14 mg GAE total phenolic content (TPC). Total antioxidant capacity (TAC) in BP was of 40.29 ± 1.24 mM TE/g (Table 2).

### 3.3. The Elemental Composition of the Fish Muscles

Analysis of the muscular tissue of African catfish fed BP-supplemented diets did not show statistically significant differences in Zn, Mn, Fe, K, Na and Mg levels in comparison to their levels in the control group. However, statistically significant differences were observed in Ca levels among the studied groups (Table 3). The muscular tissue of African catfish fed with 1 and 2% BP contained statistically more (group BP1: 147 mg/kg, group BP2: 178 mg/kg) of Ca than the control group (77 mg/kg). The Ca content in the muscular tissue of fish from the BP3 group (102 mg/kg) did not differ statistically from the BP1 and C groups (Table 3). The highest Mn (0.29 mg/kg), Fe (3.57 mg/kg), and Mg (317 mg/kg) contents were observed in the tissue of fish from the BP3 group, while Ca (177.68 mg/kg), Zn (19.40 mg/kg) and Na (508.35 mg/kg) in BP2 fish (Table 3).

### 3.4. Intestinal Microflora

**Figure 1:**
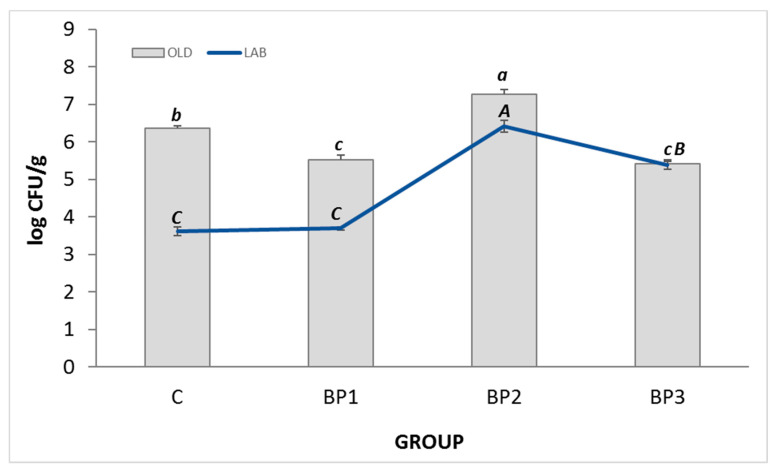
Total microbial count (OLD) and lactic acid bacteria count (LAB) in the gut of juvenile African catfish *Clarias gariepinus* fed commercial feed without (C), and with 1% (BP1), 2% (BP2), 3% (BP3) honeybee pollen. Groups marked with different letters are statistically significant (*p* < 0.05; *Tukey test*).

Statistically, the highest total microbial count was found in group BP2 (7.27 log CFU/g, colony-forming unit), while the lowest was in groups BP1 and BP3 (5.51 log CFU/g and 5.41 log CFU/g) (Figure 1). The number of LAB was the highest in the BP2 group and amounted to 6.42 log CFU/g, and the lowest in the control group (group C; 3.61 log CFU/g) and the BP1 group (3.69 log CFU/g) (Figure 1). In the intestinal content of fish from the control group C, 57% of the total number of microorganisms were LAB. In research groups BP1 and BP2, the number of microorganisms accounted for 67 and 88% of the total number of microorganisms, respectively. In the BP3 group, almost 100% of the intestinal microbiota composition was LAB, which was a statistically significant value compared to the other groups. The MALDI-TOF analysis showed that LAB strains of the genus *Lactococcus garvieae* prevailed in the control group. In the groups fed BP-supplied diets (BP1, BP2, and BP3 groups), apart from *L. garvieae*, the presence of *L. lactis* and *Bacillus amyloliquefaciens* subsp. *plantarum* was found.

### 3.5. Histological Analysis

#### 3.5.1. Intestinal Histomorphology and Morphometry

The histological analysis showed that the intestinal wall of African catfish fed with BP-enriched diets was thicker than that of fish from the control group. The width of the intestinal wall was statistically significantly thicker in the groups fed BP-based diets compared to the control group (Table 4). The thickest intestinal wall was reached by fish from BP2 and BP3 groups, 83.4 and 83.5 µm, respectively. The height of the intestinal villi of African catfish from groups BP1 (471.5 µm), BP2 (532.5 µm) and BP3 (492.7 µm) was statistically significantly higher (*p* < 0.05) than in group C (387.5 µm). Statistically significantly higher enterocyte height was found in fish from the BP1 group (34.9 µm) compared to fish from C, BP2 and BP3 groups. 

**Figure 2:**
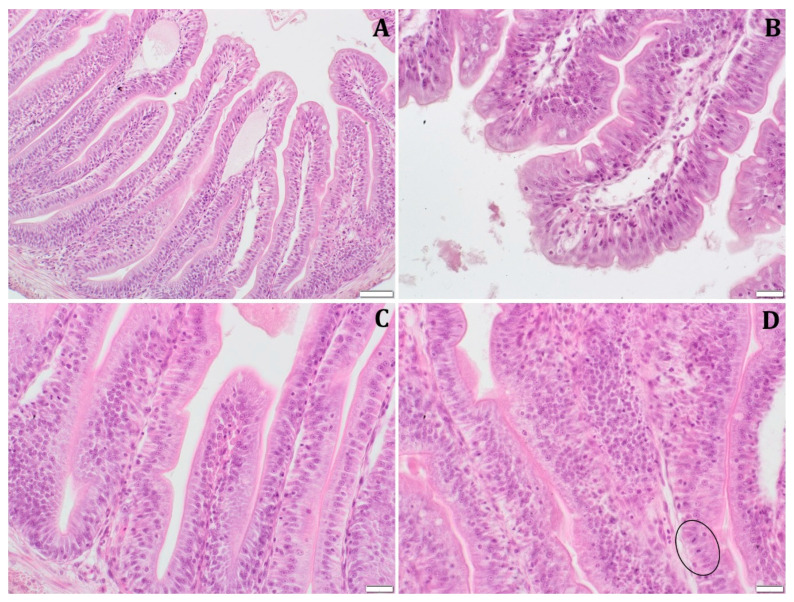
Histological photomicrograph of the midgut of juvenile African catfish *Clarias gariepinus* juveniles fed commercial feed without the addition of honeybee pollen (group C: **A**) and with the addition of 1% (BP1: **B**), 2% (BP2: **C**), 3% (BP3: **D**) honeybee pollen. The loop indicates mitotic activity (scale bar: 50 µm—(**A**); 10 µm—(**B**), 20 µm—(**C**,**D**)).

The histopathological analysis also showed no significant differences in the structure and appearance of the intestinal villi. There was slight swelling of the intestinal villi and dilation of the lymphatic vessels in the control group (Figure 2A). Within the research groups, the presence of single foci of enterocyte hyperplasia was observed. In addition, in the groups fed BP-supplied diets, there were foci of the proliferation of enterocytes with single mitotic figures (Figure 2B–D).

#### 3.5.2. Hepatic Histoarchitecture

**Figure 3:**
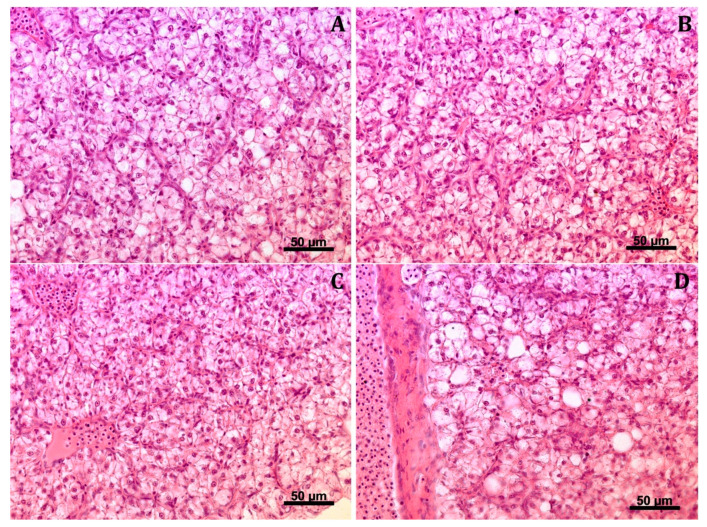
Histological photomicrograph of the hepatic tissue of juvenile African catfish *Clarias gariepinus* fed commercial feed without the addition of honeybee pollen (group C: **A**) and with the addition of 1% (BP1: **B**), 2% (BP2: **C**), 3% (BP3: **D**) honeybee pollen (scale bar: 50 µm).

The histopathological evaluation showed normal liver structure in fish from all groups. One large lipid vacuole was found in the cytoplasm of hepatocytes (Figure 3A–D). In the case of groups C and BP1, the occurrence of multifocal clusters of small lymphocytes, often located perivascular, was observed. In fish from groups BP2 and BP3, fewer clusters of lymphocytes were found in the liver parenchyma compared to fish from groups C and BP1.

## 4. Discussion

Improving and optimizing fish rearing is one of the priorities of modern aquaculture, as this will result in several measurable economic benefits [49]. Therefore, many research works have concentrated on increasing survival, the growth rate, or the disease resistance of farmed fish [50,51,52,53]. Interestingly, the use of functional bioactive substances in fish diets, such as herbal additives, prebiotics, vitamins, and others, has been proven to positively affect many aspects of fish culturing [50,51,54,55,56,57]. One such substance is honeybee pollen (BP), which was tested in the breeding of juvenile stages of African catfish in this work.

To put it briefly, it was found that feeding juvenile African catfish with diets supplied with either 1%, 2%, or 3% BP resulted in a significant increase in rearing parameters, such as body weight and length, and the SGR coefficient. Moreover, no significant effects of BP-supplemented diets on the fish survival rate were observed, although survival in the control group was about 10% lower than in the study groups. However, considering the intestinal microbiota, it can be concluded that the addition of BP to the feed had a positive effect on the development of several LAB-probiotic microorganisms in the intestinal tract of the treated fish. The results of this work indicate that BP can be successfully used as a feed additive in the feeding strategies of African catfish juveniles. The dietary addition of BP positively affected fish growth and related indicators. The authors came to similar conclusions when testing the addition of BP in the feeding rainbow trout [38,39] and Nile tilapia [37]. However, in the case of Nile tilapia, fish fed with feed containing BP at a concentration of 2.5% had significantly higher final weight, body length, and faster growth rate than fish from the control group [37]. Differently, BP-based diets did not significantly affect the nutrient utilization and growth performance of meagre juveniles [40]. These discrepancies might be associated with fish species differences, feeding habits, intestinal structure, feeding duration, experimental design, or others. It can also be assumed that the higher growth parameters achieved, and at the same time, the higher survival rate were the result of the simultaneous effect of bioactive compounds contained in honeybee pollen, given in the feed to juvenile African catfish. The results of studies described by other authors indicate that in the case of adding bee pollen or bee pollen extract to the feed, better results of growth, survival, intestinal development and intestinal microbiome, as well as increased immunity and stress response are often achieved e.g., [23,24,25,34,35,36,37,38,39]. It also allows for better use and digestion of the feed [40], and thus has a positive effect on the parameters described above.

There are several reasons for the fish growth improvement such as (a) the composition and the beneficial ingredients present in BP such as minerals, vitamins, enzymes, and co-enzymes, which will, in turn, help to improve the process of digestion and nutrient assimilation [58,59], (b) increased the intestinal surface area of absorption, as occur in our study, via increasing the length and thickness of the intestinal villi in broilers [59] and rats [60] that fed BP-supplemented diets, and (c) improvement of protein anabolism in animals [61]. Although the above-mentioned factors, the actual precise roles of BP in improving fish growth require further research studies. Its effects on the expression of growth-related genes also merit additional investigations. The present study showed no significant effects of BP supplementation on the chemical composition of the fish body; however, an upward trend was found in the amount of some elements (e.g., Fe, Na, K, and Mn). Total survival rates were also about 9% higher in the BP-supplemented groups (BP1, BP2, and BP3) compared to the control group, although these values were not statistically significant. However, raising these important parameters may affect the economic benefits of breeding. It was previously reported that BP is not only an important source of many vitamins, amino acids, and fatty acids [62,63] but also polyphenols, including flavonoids, which have strong antioxidant properties [64]. This study showed that the honey BP, used in this study, contained total phenolic content 28.14 ± 0.23 mg GAE/g, including 19.64 ± 0.58 mg QE/g flavonoids. An antioxidant BP capacity was 40.29 ± 1.24 mM TE/g. These data are important for the body’s defense against free radicals and pathogenic agents. Polyphenols also have potent antioxidant, antibacterial and antifungal activities [29,62,65,66].

The reduction of pathogenic microorganisms present in the intestines may favor the development of probiotic microflora [56,67]. In this study, the total count of the intestinal microbiota of African catfish was reduced due to the BP-supplied diets. The present study showed that BP-supplied diets significantly impacted the intestinal microbiota of African catfish by increasing the percentage of LAB, which have a potential probiotic effect [56]. The MALDI-TOF analysis showed that in fish groups fed BP-supplied diets, the intestinal microflora was LAB, such as *L. latis*, *B. amyloliquefaciens* subsp. *plantarum* or *Lactococcus* ssp. In the animal feed production industry, LAB is mainly used as probiotics to improve animal health and their productive capacity [53,68]. Research studies performed by Reda et al. [69] showed that *B. amyloliquefaciens* bacteria could improve the growth, disease resistance, and hematology indices of African catfish. Probiotic microorganisms can also increase fish’s resistance to pathogen infections [70]. Not without significance is also the increase in resistance to bacterial infections as a result of feeding honeybee pollen to fish, which directly affects the health of fish, and thus the obtained breeding results e.g., [24,27,29,37,39,40]. Research by Di Chiacchio et al. [25] also indicate the possibility of transferring increased immunity to offspring, which potentially increases the possibility of using honeybee pollen in aquaculture.

BP could also be used as a prebiotic with unique characteristics. For instance, El-Asely et al. [37] showed that tilapia fed with 2.5% BP had the highest protection against *Aeromonas hydrophila* infection compared to control fish. Additionally, in the case of gilthead sea bream, it significant increase was found in the bactericidal activity against *Vibrio harveyi* in the groups fed BP-supplemented diets with respect to the control groups [33]. The BP effects on increasing the number of probiotic microorganisms were also found in poultry [59,71]. Moreover, the BP effects on increasing the number of probiotic bacteria of the genus *Lactobacillus* spp. and *Enterococcus* spp. was also formerly demonstrated [71], and the development of the intestine by increasing the absorption of nutrients [59]. As a result, higher weight gains of chickens fed with feed with the BP addition were observed in compared to poultry from the control group. Therefore, the findings obtained in our study could support the new challenge of the food industry as it could focus on producing antibiotic-free foods, and the evidence suggests that the use of LAB in production systems could represent one of the most important tools in the pursuit of this achievement [66]. What is particularly interesting, these changes were observed after only three weeks of rearing of African catfish fry. This confirms the earlier observations of many authors that when rearing larvae and fry in optimal thermal conditions, a period of 2–3 weeks is sufficient to identify possible changes or differences between the groups both in the internal organs and in the intestinal microbiome [52,72,73,74,75,76,77].

Prebiotics such as BP can also benefit intestine development and health [51]. In the present study, fish-fed BP-based diets were found to have significantly higher intestinal villi and wall thickness than control fish. In addition, there was hyperplasia of the intestinal villi and slight hyperemia of the blood vessels. The presence of foci of enterocyte hyperplasia and the higher average height of intestinal villi may suggest faster and earlier maturation of the intestines, which may translate into increased feed assimilation [59]. The blood and lymphatic vessels in the intestinal villi carry the digested food into the body fluids. These findings may also suggest that dietary BP may positively affect the development of the intestinal and better absorption of nutrients. Nonetheless, the precise effects of BP require a complete profile of the entire intestinal microbiome. Thus, additional research studies should be undertaken.

## 5. Conclusions

The addition of honeybee pollen in the feed had a positive effect on the rearing of African catfish fry. Significant differences in growth, gut development and the composition of the gut microbiome were observed after only three weeks of rearing of African catfish fry. The addition of BP in the nutrition strategies of juvenile African catfish also may induce a positive effect on the development of the intestinal tract manifested by increasing the surface area of absorption in the intestine. Considering the economic aspects of the dietary supplementation of BP to feed intended for African catfish breeding, it can be concluded that supplementation in the amount of 1% BP will be justified. Because of beneficial effects BP, maybe used as a functional feed ingredient for diets of African catfish juveniles with a significant prebiotic effect. Although the effects of dietary BP, its effects on the immunity, expression of immune-related genes, disease resistance, and identification of the entire gut-associated microbiome of African catfish warrants additional investigations.

It seems very interesting in the future to investigate how long the positive effects of administering the additive in honeybee pollen feed will last. This would allow periodic administration of this additive in the feed, e.g., before sorting fish, which would reduce stress, increase immunity, etc. ... and thus should have a positive impact on the final aquaculture effects.

## Figures and Tables

**Table 1:** Results (mean ± SD) of rearing of African catfish *Clarias gariepinus* juveniles under controlled conditions and fed with commercial feed (group C) and those feed with the addition of 1% (group BP1), 2% (group BP2) and 3% (group BP3) honeybee pollen.

Measured Parameters	Experimental Groups			
	C	BP1	BP2	BP3
Initial weight (g)	0.48 ± 0.17	0.48 ± 0.17	0.48 ± 0.17	0.48 ± 0.17
Initial length (mm)	38.9 ± 4.5	38.9 ± 4.5	38.9 ± 4.5	38.9 ± 4.5
Final weight (g)	5.01 ± 2.05 ^b^	7.38 ± 2.92 ^a^	6.63 ± 1.84 ^a^	7.45 ± 3.17 ^a^
Final length (mm)	84.7 ± 9.7 ^b^	95.9 ± 12.1 ^a^	93.8 ± 8.4 ^a^	95.2 ± 12.8 ^a^
Growth (g/d)	0.23 ± 0.02 ^b^	0.34 ± 0.05 ^a^	0.31 ± 0.03 ^a^	0.35 ± 0.05 ^a^
Specific growth rate (%/d)	11.70 ± 0.45 ^b^	13.63 ± 0.64 ^a^	13.11 ± 0.30 ^a^	13.68 ± 0.65 ^a^
Condition factor	0.82 ± 0.04	0.83 ± 0.02	0.81 ± 0.03	0.86 ± 0.06
Relative growth rate (%)	941.33 ± 91.69 ^b^	1436.76 ± 196.29 ^a^	1277.30 ± 82.60 ^a^	1451.96 ±193.72 ^a^
Cumulative natural mortality (%)	11.3 ± 3.1	6.0 ± 5.3	5.3 ± 2.3	6.0 ± 3.5
Cannibalism factor (%)	10.0 ± 2.0	6.0 ± 2.0	6.7 ± 1.2	6.7 ± 4.6
Total survival rate (%)	78.7 ± 4.6	88.0 ± 3.5	88.0 ± 2.0	87.3 ± 4.2

Data in row marked with different letters are statistically significant (*p* < 0.05; *Tukey’s test*).

**Table 2:** Total antioxidant capacity (TAC), total flavonoid content (TFC) and total phenolic content (TPC) in honeybee pollen used to enrich the feed of juvenile African catfish *Clarias gariepinus*.

TAC			TFC	TPC
mM TE/g	mg TE/g	IC_50_ mg/mL	mg QE/g	mg GAE/ g
40.29 ± 1.24	10.27 ± 0.33	1.87 ± 0.07	19.64 ± 0.58	28.14 ± 0.23

**Table 3:** Levels of elements (mean ± SD) found in the muscular tissue of African catfish *Clarias gariepinus* juveniles reared under controlled conditions and fed with commercial feed (group C) and those feed with the addition of 1% (group BP1), 2% (group BP2) and 3% (group BP3) honeybee pollen.

Analyzed Elements (mg/kg)	Experimental Groups			
	C	BP1	BP2	BP3
Ca	77.13 ± 64.56 ^c^	146.80 ± 30.98 ^ab^	177.68 ± 43.6 ^a^	102.30 ± 19.99 ^bc^
Zn	12.3 ± 2.60	12.42 ± 2.28	19.40 ± 14.32	11.87 ± 1.37
Mn	0.15 ± 0.15	0.19 ± 0.16	0.19 ± 0.11	0.29 ± 0.16
Fe	2.86 ± 0.60	2.73 ± 0.76	3.06 ± 1.08	3.57 ± 1.93
K	2871.75 ± 145.46	3010.25 ± 52.90	2952.25 ± 102.59	2980.25 ± 130.23
Na	498.58 ± 55.32	464.50 ± 65.60	508.35 ± 45.59	507.20 ± 22.72
Mg	293.48 ± 22.16	305.55 ± 7.06	288.60 ± 18.11	317.00 ± 10.17

Data in row marked with different letters are statistically significant (*p* < 0.05; *Tukey’s test*).

**Table 4:** Intestinal histomorphometric measurements of juveniles African catfish reared in controlled conditions and fed with commercial feed (group C) and those fed a diet supplemented with 1% (BP1), 2% (BP2), 3% (BP3) honeybee pollen.

Parameters (µm)	Experimental Groups			
	C	BP1	BP2	BP3
Villi height	387.46 ± 83.17 ^b^	471.50 ± 63.90 ^a^	532.48 ± 67.34 ^a^	492.73 ± 110.22 ^a^
Villi width	71.88 ± 11.69	76.61 ± 9.18	73.29 ± 10.48	72.04 ± 12.29
Enterocytes height	28.94 ± 3.28 ^b^	34.85 ± 6.33 ^a^	31.83 ± 6.06 ^b^	30.20 ± 9.84 ^b^
Width of intestinal wall	67.51 ± 20.95 ^b^	76.85 ± 12.80 ^ab^	83.38 ± 24.67 ^a^	83.48 ± 19.78 ^a^

Values in rows with different letters are statistically significant (*p* < 0.05; *Tukey test*).

## Data Availability

The data presented in this study are available on request from the corresponding author. The data are not publicly available due to privacy.
